# Hybrid networks based on epoxidized camelina oil

**DOI:** 10.1080/15685551.2016.1231031

**Published:** 2016-09-16

**Authors:** Brindusa Balanuca, Raluca Stan, Adriana Lungu, Eugeniu Vasile, Horia Iovu

**Affiliations:** ^a^ Department of Bioresources and Polymer Science, Advanced Polymer Materials Group, University Politehnica of Bucharest, Bucharest, Romania; ^b^ Department of Organic Chemistry, University Politehnica of Bucharest, Bucharest, Romania; ^c^ Academy of Romanian Scientists, Bucharest, Romania

**Keywords:** Epoxidized camelina oil, POSS, nanocomposites, reinforcements

## Abstract

Lately, renewable resources received great attention in the macromolecular compounds area, regarding the design of the monomers and polymers with different applications. In this study the capacity of several modified vegetable oil-based monomers to build competitive hybrid networks was investigate, taking into account thermal and mechanical behavior of the designed materials. In order to synthesize such competitive nanocomposites, the selected renewable raw material, camelina oil, was employed due to the non-toxicity and biodegradability behavior. General properties of epoxidized camelina oil-based materials were improved by loading of different types of organic-inorganic hybrid compounds – polyhedral oligomeric silsesquioxane (POSS) bearing one (POSS1Ep) or eight (POSS8Ep) epoxy rings on the cages. In order to identify the chemical changes occurring after the thermal curing reactions, FT-IR spectrometry was employed. The new synthesized nanocomposites based on epoxidized camelina oil (ECO) were characterized by dynamic mechanical analyze and thermogravimetric analyze. The morphology of the ECO-based materials was investigate by scanning electron microscopy and supplementary information regarding the presence of the POSS compounds were establish by energy dispersive X-ray analysis and X-ray photoelectron spectroscopy. The smooth materials without any separation phase indicates a well dispersion of the Si–O–Si cages within the organic matrix and the incorporation of this hybrid compounds into the ECO network demonstrates to be a well strategy to improve the thermal and mechanical properties, simultaneously.

## Introduction

Vegetable oil represents the most important renewable raw material used in macromolecular field in order to replace the petroleum-based compound.[1–3] Due to the environmental requirements and considering depletion of fossil resources as a warning, design of some new strategies to synthesize competitive products derived from renewable resources for new polymeric materials with superior properties represents a necessity in this area.[4–6]

In the last years, many studies report the use of vegetable oils derivatives to obtain polymeric and composite materials.[7–16] Recently linseed and soybean oils were considered the most popular triglycerides due to their availability and low price.[17] For example, epoxidized linseed oil was reinforcing with inorganic nanosized clusters to obtain materials with promising properties.[18]

In the current study we have chosen an underexploited oil, camelina oil (CO), to design several materials, taking into account the CO advantage regarding the fatty acids profile against other vegetable oils like soybean or linseed oil and also the price, abundance and facile acquire.[19–21] The used CO is characterized by a well amount of monounsaturated acids (oleic, cis 11-eicosenoic, and erucic acid) and low saturated fatty acid content [22] and thus a great functionalization degree can be achieved. By extrapolating the theory of the better functionalization with the higher monounsaturated acid content,[23] probably the epoxy rings from these kinds of acids are easily to attack during the curing reactions, due to the reduce steric hindrance and a easier penetration of the crosslinker and initiator, in comparison with the polyunsaturated acids.

Thus, current work involves epoxidized camelina oil (ECO) to design novel hybrid materials using different types of functionalized polyhedral oligomeric silsesquioxanes (POSS) reinforcing agents. Phthalic anhydride (PA) was employed as cross-linking agent according to several studies reported in literature and the ring opening reaction was catalyzed by an imidazole.[24,25] The use of both PA and imidazole was considered due to the low reactivity of the internal epoxy rings from the triglyceride chains.[6] In this way, hybrid nanocomposites were synthesized using ECO as monomer to build the epoxy matrices. From our knowledge, the use of CO-based epoxides to obtain hybrid networks has not been reported elsewhere.

## Experimental section

### Materials

CO was kindly supplied by University of Agricultural Sciences and Veterinary Medicine Bucharest being obtained by cold-pressing extraction. The curing agent: PA and the initiator: 1-methyl imidazole (1-MeI) were purchased from Sigma-Aldrich and used as received, without further purification.

**Figure 1.**
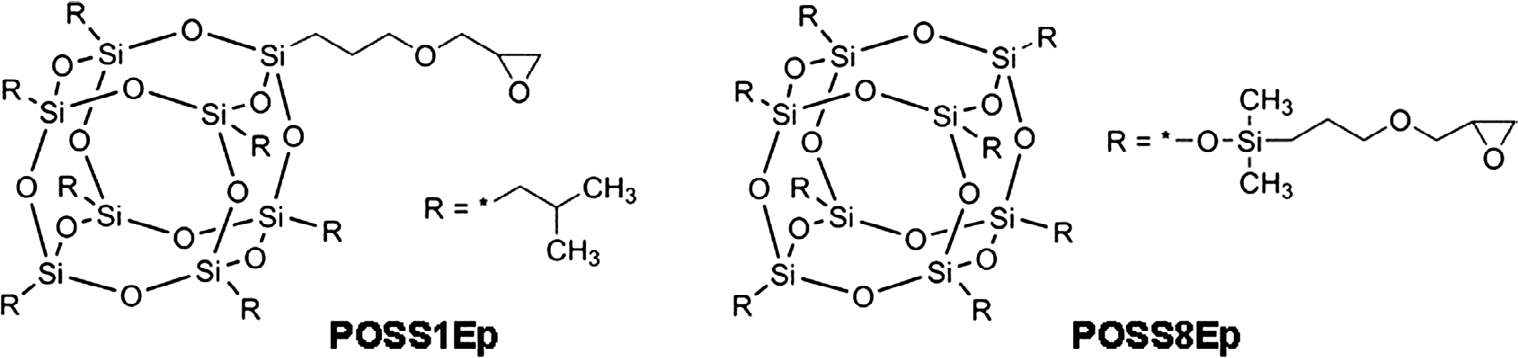
POSS derivatives chemical structures.

PSS-(3-Glycidyl) propoxy-Heptaisobutyl substituted (POSS1Ep) and PSS-Octa [(3-glycidyloxypropyl) dimethylsiloxy] substituted (POSS8Ep) derivatives, with chemical structure depicted in Figure 1 were also received from Sigma-Aldrich.

As extractive solvent for gel fraction (GF) determination, tetrahydrofuran received from Sigma-Aldrich was used. All other solvents and reagents were supplied by Sigma-Aldrich and used as received.

### Methods

ECO was synthesized by chemical modification of the triglyceride involving double bonds conversion into epoxy rings, using a molar ratio H_2_O_2_: glacial acetic acid: CO unsaturation of 10:2:1, according to previously reported work for both CO [22] and for linseed oil.[26]

A procedure for cross-linking of the epoxy resins has been carried out using PA as cross-linking agent and 1-MeI as catalyst. The formulations were prepared using a molar ratio epoxy groups: PA of 1:1.[6] Due to the fact that PA is insoluble in ECO, a certain amount of methyl ethyl ketone (MEK) was added to improve the solubility.

The mixture of PA and MEK (5 mL) was subjected to magnetic stirring for 1 h at room temperature. The epoxidized oil and 2% wt 1-MeI were then added and the magnetic stirring is maintained for another 30 min to complete mixing of the components. The homogeneous mixture was kept for 24 h in a vacuum oven to evaporate the solvent.

To obtain reinforced networks, 5% wt POSS1Ep and respectively POSS8Ep were added over the evaporated mixtures, formulated as described above and they were sonicated 30 min to achieve a good dispersion of the POSS nanocages within the polymeric matrix.

All the synthesized samples were cast in clean teflon molds (10 mm wide, 2 mm thick and 20 mm long), cured 2 h at 120 °C and post-cured 1 h at 150 °C.

### Characterization techniques

FT-IR spectra were recorded on a Bruker VERTEX 70 spectrometer equipped with an attenuated total reflectance (ATR) accessory, using 32 scans with a resolution of 4 cm^−1^ in 600–4000 cm^−1^ wave number region.

GF analysis was performed by Soxhlet extraction using tetrahydrofuran as solvent to dissolve the soluble fraction of the materials. The weight of the cured samples was firstly measured and then each one was extracted with THF for 24 h to determine the insoluble part in the samples. After extraction the materials were dried in a vacuum oven (40 °C, 24 h) and their final masses were recorded.

Dynamic mechanical properties of the nanocomposites were measured using a Tritec 2000 (Triton Technology) instrument operated in single cantilever bending mode. The rectangular samples with approximate dimensions of 10 mm wide, 2 mm thick, 20 mm long were tested at 1 Hz in a temperature range of 25–200 °C at a heating rate of 5 °C/min.

Thermo-gravimetric curves were registered on a Q 500 TA equipment. Samples about 2.5 mg were heated from 25 to 600 °C at a heating rate of 10 °C/min under constant nitrogen flow.

A scanning electron microscope (SEM Quanta Inspect F) was used to study the morphology of hybrid materials. The specimens were manually broken allowing cross-sectional analysis. Prior to be analyzed the samples were spray coated with a thin layer of gold to enhance the surface conductivity. Energy dispersive X-ray (EDX) analysis was also used to identify the presence of the reinforcing agents within the hybrid networks.

X-ray photoelectron spectra were recorded on Thermo Scientific K-Alpha equipment, fully integrated, with an aluminum anode monochromatic source. Survey scans (0–1200 eV) were performed to identify constitutive elements (C1s, O1s, and Si2p).

## Results and discussion

The functionalization pathway to produce ECO and also the characterization of the obtained compound was recently described by our group.[22,26,27] In these studies the epoxidized compound (ECO) acts as an intermediary product, being used to synthesize a methacrylate functionalized oil.

**Figure 2.**
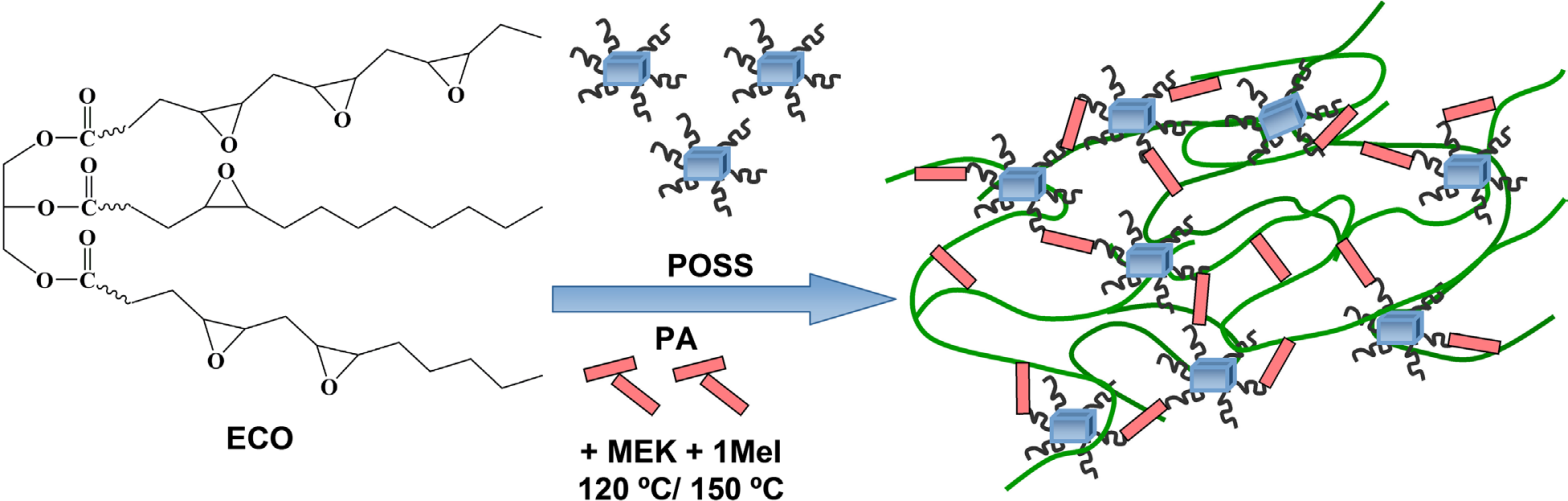
Cross-linking reactions to produce ECO-based nanocomposites.

The obtained ECO was involved to design some new materials for which the oil-based monomer represents the natural component. Moreover, a series of ECO-based hybrid materials were synthesized using POSS compounds as reinforcing agents, in order to improve the general properties. Figure 2 presents a schematic view of the cross-linking process and the obtained final hybrid material.

### FT-IR spectroscopy

**Figure 3.**
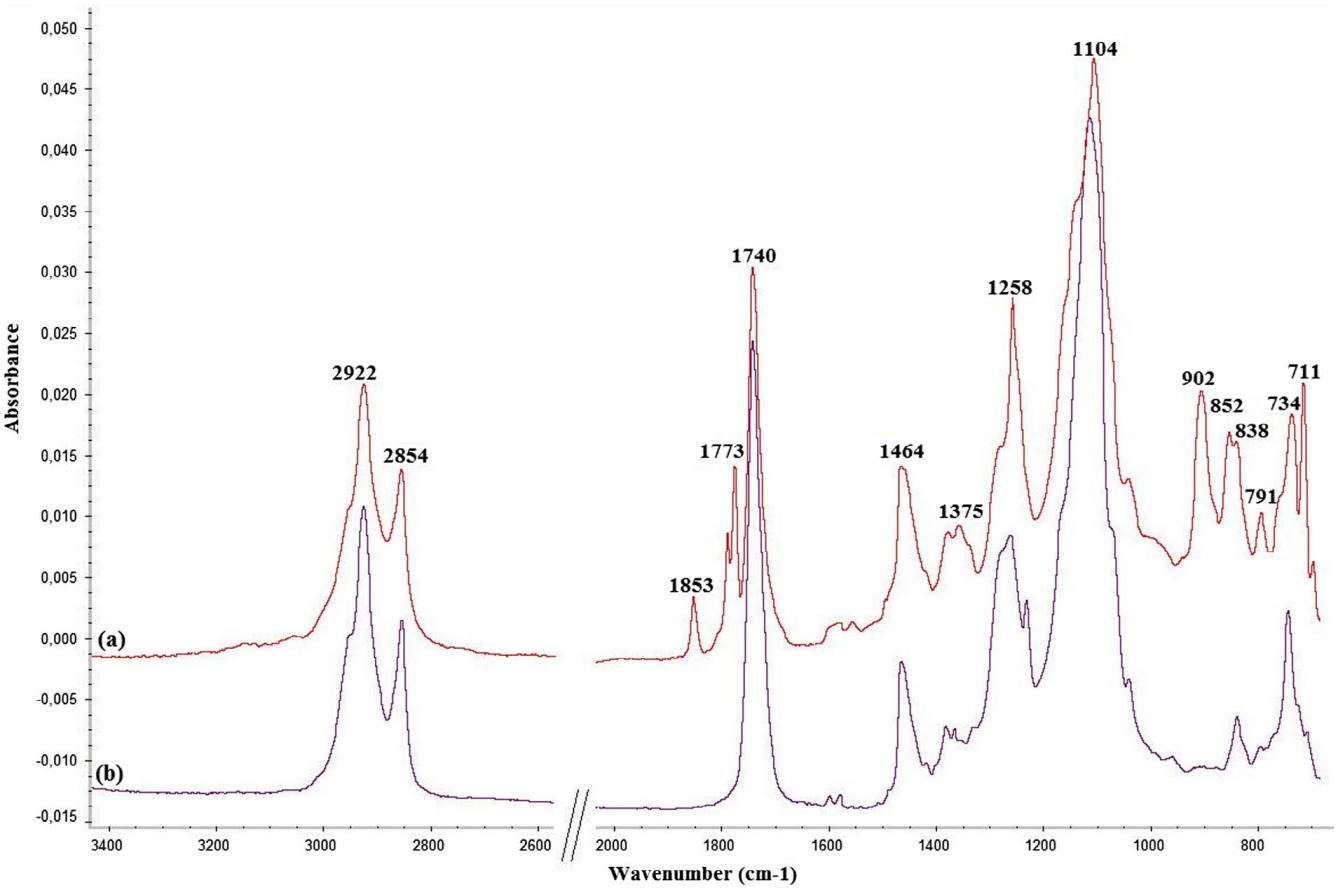
FT-IR spectra of ECO_POSS1Ep: (a) before and (b) after curing.

Prior to curing reaction, FT-IR spectra were registered for all formulated systems (Figure 3(a) – ECO_POSS1Ep system before curing procedure).

*FTIR* (ATR, cm^−1^)*:* 2922, 2854 (ν_C–H_ asim, sim.); 1853, 1773 (asymmetric and symmetric ν_C=O_ from PA); 1740 (ν_C=O_); 1464, 1375 (*δ*_CH_ from CH_2_ and CH_3_); 1258, 1104 (ν_C–O_); 902-791 (ν_C–O–C_ from epoxy ring); 734-711 ($\rho_{{\text{CH}}_{2}}$).

The crosslinking of the epoxy groups is clearly confirmed in the cured samples: the intensity of the absorption band at 902 cm^−1^ assigned to epoxy ring stretching vibrations significantly decreases (Figure 3(b)) and also the shifting of the characteristic bands for PA C=O bonds (1853 and 1773 cm^−1^) to 1740 cm^−1^ due to the formation of an ester group during the cross-linking process, indicates the success of the reaction under the selected conditions.

### GF analysis

The GF determination can be assumed as an index of the degree of cross-linking. Therefore, the GF of the synthesized materials can be calculated using Equation (1),[28] where *w*_0_ are initial weight of the cured sample and *w*_1_ represents the final weight (the insoluble part, after extraction).

(1) $$GF\%=\frac{w_{1}}{w_{0}}\times 100$$

The calculated GF values indicate high cross-linking degrees for all the cured samples (over 70%) which increase whit the POSS loading, the hybrid network containing POSS8Ep recording the maximum conversion degree (84%) due to the multiple covalent bonds formed by this cage with the organic matrix.

### Dynamic mechanical analyze tests

**Figure 4.**
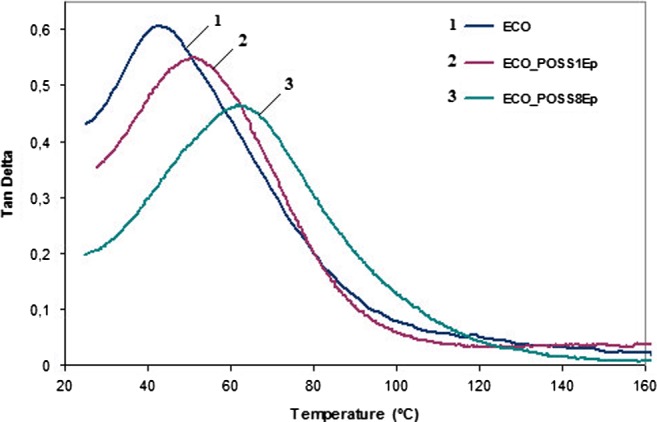
The dependence of Tan *δ* vs. temperature for ECO_POSS nanocomposites.

Dynamic mechanical analysis offers valuable indication about the networks mobility and crosslinking density of the oil-based materials. The Tan *δ* as function of temperature for the bio-based materials is graphically represented in Figure 4 and registered *T*_g_ values are presented in Table 1.

**Table 1.** Thermo-mechanical and thermal-stability results.

Sample	*T*_g_^a^(°C)	Weight loss (°C)			*T*_max_^e^	Residual mass (600 °C) (%)
		*T*_d_ 5%^b^	*T*_d_ 50%^c^	*T*_d_ 90%^d^		
ECO	45	162	355	423	368	1.2
ECO_POSS1Ep	51	272	369	422	382	1.7
ECO_POSS8Ep	64	299	384	446	391	3.0

^a^ *T*_g_ – the maximum of tan *δ* plots was considered for the glass transition temperature (*T*_g_) determination.

^b^ *T*_d_ 5% – temperature at which the mass loss of the sample is 5%.

^c^ *T*_d_ 50% – temperature at which the mass loss of the sample is 50%.

^d^ *T*_d_ 90% – temperature at which the mass loss of the sample is 90%.

^e^ *T*_max_ – temperature at which the weight loss is maximum.

Regarding the glass transition of the synthesized nanocomposites, the lower *T*_g_ value of 45 °C was registered for neat ECO, inferior than those for the cured commercial resin [27,29,30] but superior than those of other epoxidized vegetable oil-based cured systems.[24] With long fatty acids chains on the structure, neat ECO is characterized by higher flexibility reflected on low *T*_g_ in comparison with conventional epoxy resin with rigid aromatic rings.

When POSS1Ep was loaded, an improvement of the glass transition was observed which can be explained by a decrease on the material flexibility once the hybrid nanocages were added and more crosslinking points are formed. A significant increase of the *T*_g_ value at 64 °C was attained for ECO_POSS8Ep system compared with POSS-free oil based network, attributed to a higher crosslinging density achieved based on more reactive sites available for POSS8Ep, which led to a decrease of long chain motion.

### Thermogravimetric analyze results

**Figure 5.**
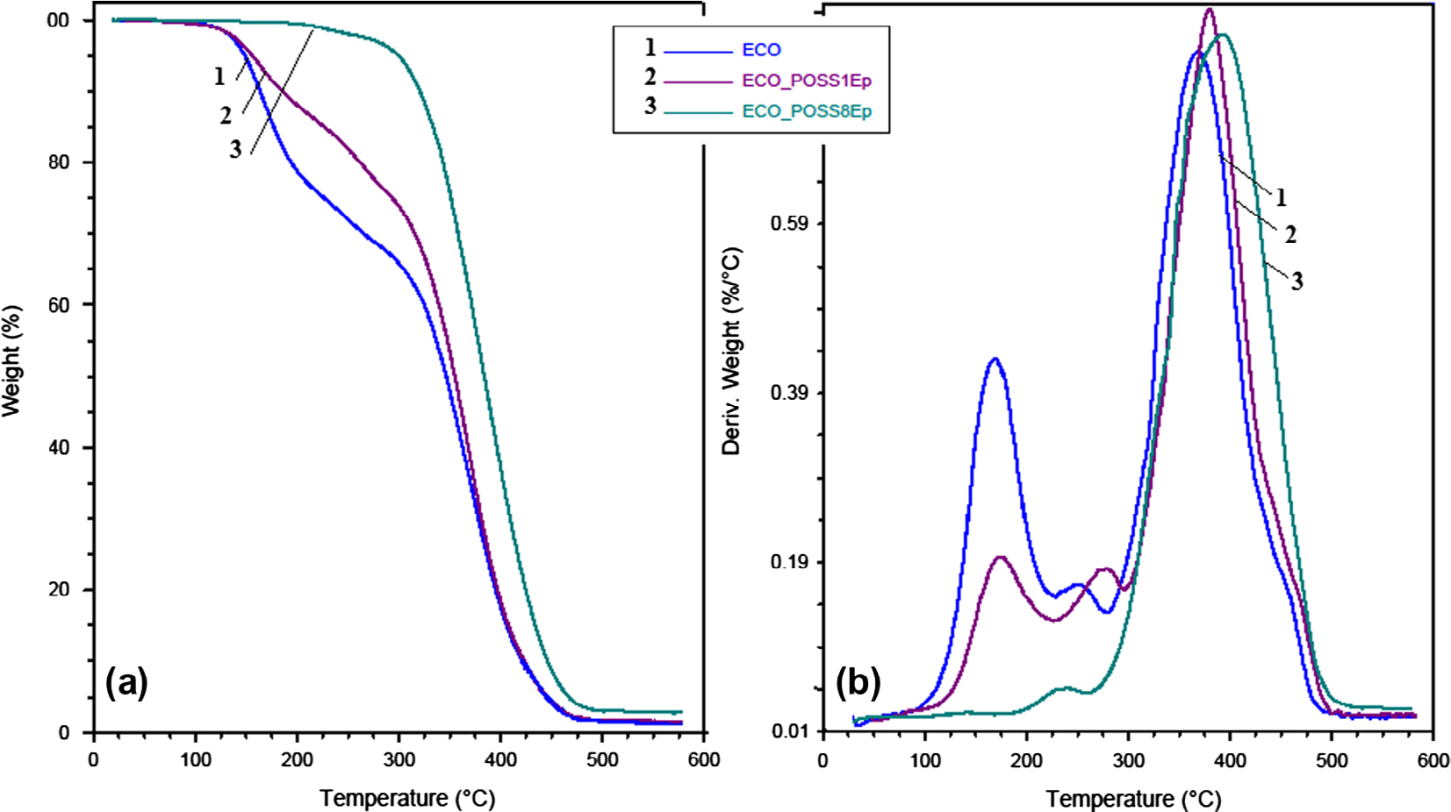
(a) TGA and (b) DTG plots of ECO system and ECO_POSS nanocomposites.

Concerning the thermal resistance of the ECO-based materials, a very good thermal stability was gained when POSS was loaded (Figure 5(a)). Thus the value of *T*_d_ 5% (Table 1) increased significantly for ECO_POSS1Ep in comparison with neat ECO.

If the hybrid was obtained by reinforcing of ECO resin with POSS8Ep, 5% weight loss occurs at a much higher temperature (299 °C). This behavior may be explained by the multiple cross-linking points formed by the octa-functional POSS with ECO resin which means that a network with high molecular mass is achieved so that low molecular weight fractions are not probably to occur.[31]

At higher temperatures (400 °C) the difference in the behavior of ECO and ECO_POSS nanocomposites is not very significant since the degradation processes follows the same trend (Table 1). Also, thermogravimetric analyze results indicate an increased residual mass for the reinforced networks, due to the presence of the inorganic component.

Regarding thermal decomposition of the designed materials the cured ECO and ECO_POSS1Ep samples exhibit three decomposition peaks. When POSS8Ep is used to reinforce the ECO-based network, one principal degradation step is registered. The thermal stability of ECO_POSS8Ep is attributed to a higher crosslinking density, only a small fraction of low molecular weight being probably formed (reflected to the small shoulder registered to the DTG trace – Figure 5(b)). This behavior and also the higher temperature associated to the maximum decomposition rate (according to *T*_max_) are strong arguments for the role played especially by POSS8Ep compounds acting as a thermal stabilizer for the ECO chains.

Thermal degradation of the reinforced sample led to low residual mass due to the small amount of POSS introduced into the oil-base matrix. However difference between ECO_POSS1Ep and ECO_POSS8Ep from this point of view can be explain by the different substituents grafted on the Si–O–Si cages, POSS8Ep containing dimethylsiloxanes being able to form silica residues.

### Morphology of the ECO-based hybrids

Generally the behavior of the reinforced systems is closely related with the material morphology. To examine the morphology of the synthesized samples a magnification of 5000X was chosen.

**Figure 6.**
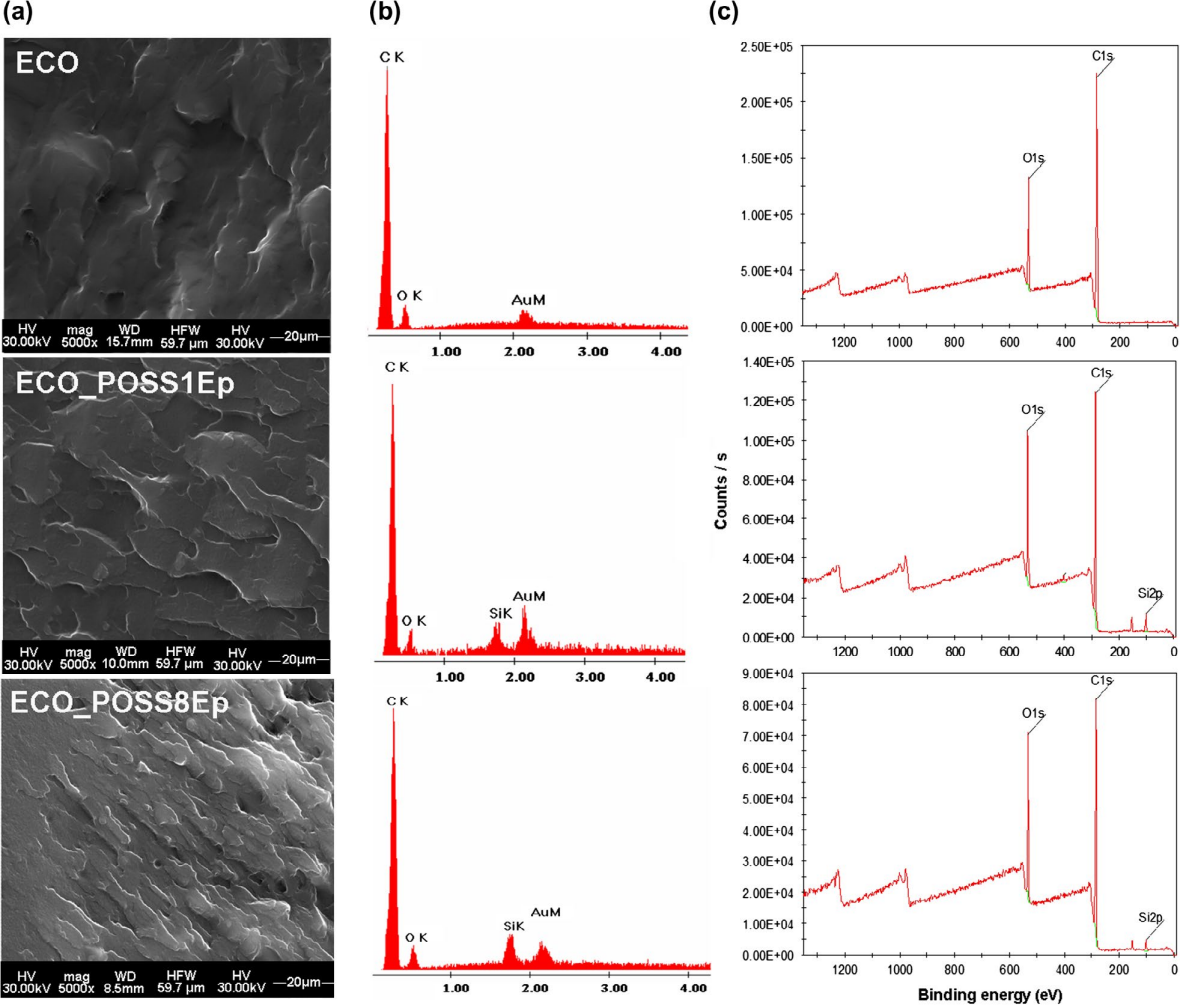
(a) SEM images, (b) corresponding EDX spectra, and (c) XPS survey spectra of the neat ECO and ECO_POSS hybrid systems.

SEM micrographs of the fractures belonging of neat ECO and hybrid systems show single phase morphology (Figure 6(a)). It can be observed small differences between fracture patterns but all ECO-based materials are microscopically homogeneous including the two types of studied hybrids, POSS compound being uniformly dispersed within the organic oil-based matrix both for one or octa-functional reinforcing agent used.

The influence of POSS components on ECO structure is pointed out by a rough appearance and this behavior is more predominant if POSS with high number of epoxy groups is loaded. In comparison with hybrid materials, neat ECO seems to be more plain, dense, and uniform. Suchlike morphological changing could be attributed to a deep intercalation of the hybrid POSS compound through the long hydrocarbonate chains of the ECO due to the higher number of the functional groups from the POSS8Ep structure allowing more cross-linking points into the oil-based matrix, in accordance with thermal and thermo-mechanical properties exhibited by the studied specimens.

Considering the same explanation of the multiple connections, ECO_POSS8Ep is characterized by a tighter packaging of the fatty acids chains which leads to the rough/bumpy aspect

SEM–EDX analysis (Figure 6(b)) was also performed to prove the POSS particles incorporation into the ECO-based matrices. For both ECO_POSS1Ep and ECO_POSS8Ep the Si–O–Si cages are uniformly dispersed, no agglomeration being observed.

X-ray photoelectron spectroscopy (XPS) analysis was performed on the surface of the fractured materials in order to prove the presence of POSS cages. The XPS spectra of ECO_POSS hybrids in comparison with neat ECO (Figure 6(c)) demonstrate the appearance of the Si (2p) and confirmed that the ECO network was successfully reinforced with Si–O–Si cages.

Taking into account the registered elements percentages (Table 2) and the chemical structure of the POSS compounds (Figure 1), the higher content of Si (2p) on the ECO_POSS1Ep system could be attributed to a specific and different ordering of the Si atoms (self-organization) from the mono-functionalized POSS due to a single possibility of coupling with the organic matrix.

**Table 2.** XPS results concerning the major constituents of the synthesized systems.

Sample	Element (at %)		
	C 1s	O 1s	Si 2p
ECO	84.12	15.88	–
ECO_POSS1Ep	77.14	18.79	4.07
ECO_POSS8Ep	77.19	19.26	3.55

Contrary, POSS8Ep is well caught on ECO matrix and the Si atoms from the Si–O–Si cages cannot self-organized because the reactive segments of this hybrid compound are linked with the other reactive sites from the system.

## Conclusions

CO-based derivative has been successfully used to design new hybrid materials. Thermal curing procedure in the presence of PA and 1-methyl imidazole as curing agent and initiator respectively was developed surpassing the problem related to the low reactivity of the internal epoxy rings from the triglyceride backbone.

The used ECO proved to be a suitable alternative in our strategy, nanocomposites with improved dynamic-mechanical properties and thermostability being obtaining by reinforcing the oil based network with different types functionalized POSS. Superior features was obtained when POSS functionalized with eight epoxy ring on the cage structure was loaded. There was finding that the POSS nanocages incorporation leads to single phase morphology and no aggregates were observed; XPS analysis demonstrate also the presence of the Si into the hybrid network.

Using CO-based epoxy derivatives we aimed to demonstrate their potential in the field of polymer and hybrid materials developing new possibilities to replace at least in part the conventional resins derived from non-renewable fossil resources with compounds from the so bidder agriculture.

The influence of POSS compounds on other physico-mechanical properties of ECO-based materials will be considered in further research.

## Disclosure statement

No potential conflict of interest was reported by the authors.

## Funding

The work has been funded by the Sectoral Operational Programme Human Resources Development 2007–2013 of the Ministry of European Funds through the Financial Agreement POSDRU/159/1.5/S/132395.

## References

[1] Miao S, Wang P, Su Z, et al Vegetable-oil-based polymers as future polymeric biomaterials. Acta Biomater. 2014;10:1692–1704. 10.1016/j.actbio.2013.08.040 24012607

[2] Xia Y, Larock RC Vegetable oil-based polymeric materials: synthesis, properties, and applications. Green Chem. 2010;12:1893–1909. 10.1039/c0gc00264j

[3] Karak N Vegetable oil-based polymers. Properties, processing and applications. UK: Elsevier Science Technology; 2012.

[4] Lligadas G, Ronda JC, Galia M, et al Renewable polymeric materials from vegetable oils: a perspective. Matter. Today. 2013;16:337–343.

[5] Islam MR, Hossen Beg MD, Jamari SS Development of vegetable-oil-based polymers. J. Appl. Polym. Sci. 2014;131. doi:10.1002/app.40787

[6] Wang R, Schuman TP Vegetable oil-derived epoxy monomers and polymer blends: a comparative study with review. Express Polym. Lett. 2013;7:272–292. 10.3144/expresspolymlett.2013.25

[7] Li S, Xia J, Xu Y, et al Preparation and characterization of acorn starch/poly(lactic acid) composites modified with functionalized vegetable oil derivates. Carbohydr. Polym. 2016;142:250–258. 10.1016/j.carbpol.2016.01.031 26917397

[8] Aranguren MI, González JF, Mosiewicki MA Biodegradation of a vegetable oil based polyurethane and wood flour composites. Polym. Test. 2012;31:7–15. 10.1016/j.polymertesting.2011.09.001

[9] Alagi P, Choi YJ, Hong SC Efficient and quantitative chemical transformation of vegetable oils to polyols through a thiol-ene reaction for thermoplastic polyurethanes. Ind. Crops Prod. 2016;87:78–88. 10.1016/j.indcrop.2016.04.027

[10] Ionescu M, Radojčić D, Wan X, et al Functionalized vegetable oils as precursors for polymers by thiol-ene reaction. Eur. Polym. J. 2015;67:439–448. 10.1016/j.eurpolymj.2014.12.037

[11] Alam M, Akram D, Sharmin E, et al Vegetable oil based eco-friendly coating materials: a review article. Arabian J. Chem. 2014;7:469–479. 10.1016/j.arabjc.2013.12.023

[12] Galia M, Montero de Espinosa L, Ronda JC, et al Vegetable oil-based thermosetting polymers. Eur. J. Lipid Sci. Technol. 2009;111:1–10.

[13] Konwar U, Karak N Hyperbranched polyether core containing vegetable oil-modified polyester and its clay nanocomposites. Polym. J. 2011;43:565–576. 10.1038/pj.2011.19

[14] Al-Mulla EAJ Polylactic acid/epoxidized palm oil/fatty nitrogen compounds modified clay nanocomposites: preparation and characterization. Korean J. Chem. Eng. 2011;28:620–626. 10.1007/s11814-010-0373-6

[15] Pelletier H, Belgacem N, Gandini A Acrylated vegetable oils as photocrosslinkable materials. J. Appl. Polym. Sci. 2006;99:3218–3221. 10.1002/(ISSN)1097-4628

[16] Fertier L, Koleilat H, Stemmelen M, et al The use of renewable feedstock in UV-curable materials – a new age for polymers and green chemistry. Prog. Polym. Sci. 2013;38:932–962. 10.1016/j.progpolymsci.2012.12.002

[17] Fiore V, Valenza A Advanced fibre-reinforced polymer (FRP) composites for structural applications. Cambridge, UK: Elsevier B.V.; 2014.

[18] Lligadas G, Ronda JC, Galià M, et al Bionanocomposites from renewable resources: epoxidized linseed oil-polyhedral oligomeric silsesquioxanes hybrid materials. Biomacromolecules. 2006;7:3521–3526. 17154483 10.1021/bm060703u

[19] Wu X, Leung DYC Optimization of biodiesel production from camelina oil using orthogonal experiment. Appl. Energy. 2011;88:3615–3624. 10.1016/j.apenergy.2011.04.041

[20] Moser BR, Vaughn SF Evaluation of alkyl esters from Camelina sativa oil as biodiesel and as blend components in ultra low-sulfur diesel fuel. Bioresour. Technol. 2010;101:646–653. 10.1016/j.biortech.2009.08.054 19740653

[21] Fröhlich A, Rice B Evaluation of Camelina sativa oil as a feedstock for biodiesel production. Ind. Crops Prod. 2005;21:25–31. 10.1016/j.indcrop.2003.12.004

[22] Balanuca B, Lungu A, Hanganu A, et al Hybrid nanocomposites based on POSS and networks of methacrylated camelina oil and various PEG derivatives. Eur. J. Lipid Sci. Technol. 2014;116:458–469. 10.1002/ejlt.201300370

[23] La Scala J, Wool RP The effect of fatty acid composition on the acrylation kinetics of epoxidized triacylglycerols. J. Am. Oil Chem. Soc. 2002;79:59–63. 10.1007/s11746-002-0435-4

[24] Gerbase AE, Petzhold CL, Costa APO Dynamic mechanical and thermal behavior of epoxy resins based on soybean oil. J. Am. Oil Chem. Soc. 2002;79:797–802. 10.1007/s11746-002-0561-z

[25] Crandall EW, Winston M: Cross-linking reaction of an epoxy resin with phthalic anhydride In: May CA, editor. Chemorheology of thermosetting polymers. Washington, DC: ACS 1983; Vol. 227 p. 113–119.

[26] Balanuca B, Stan R, Hanganu A, et al Novel linseed oil-based monomers: synthesis and characterization, U.P.B. Sci. Bull., Series B. 2014; 76, 129–140.

[27] Balanuca B, Lungu A, Conicov I, et al Novel bio-based IPNs obtained by simultaneous thermal polymerization of flexible methacrylate network based on a vegetable oil and a rigid epoxy. Polym. Adv. Technol. 2015;26:19–25. 10.1002/pat.v26.1

[28] Wang S, Kempen DH, Simha NK, et al Photo-cross-linked hybrid polymer networks consisting of poly(propylene fumarate) and poly(caprolactone fumarate): controlled physical properties and regulated bone and nerve cell responses. Biomacromolecules. 2008;9:1229–1241. 10.1021/bm7012313 18307311 PMC2888142

[29] Souza JPB, Reis JML Thermal behavior of DGEBA (diglycidyl ether of bisphenol A) adhesives and its influence on the strength of joints. Appl. Adhes. Sci. 2013;1. doi:10.1186/2196-4351-1-6.

[30] Damian CM, Pandele AM, Andronescu C, et al Epoxy-based nanocomposites reinforced with new amino functionalized multi-walled carbon nanotubes. Fullerenes, Nanotubes, Carbon Nanostruct. 2011; 19: 197–209. 10.1080/15363831003721773

[31] Pistor V, Soares BG, Mauler RS Influence of the polyhedral oligomeric silsesquioxane n-phenylaminopropyl – POSS in the thermal stability and the glass transition temperature of epoxy resin. Polímeros Ciência e Tecnologia. 2013;23:331–338. 10.4322/polimeros.2013.039

